# Association between time of assessment within a school year and physical fitness of primary school children

**DOI:** 10.1038/s41598-024-61038-x

**Published:** 2024-05-20

**Authors:** Paula Teich, Kathleen Golle, Reinhold Kliegl

**Affiliations:** Division of Training and Movement Science, Research Focus Cognitive Sciences, University of Potsdam, Am Neuen Palais 10, Building 12, 14469 Potsdam, Germany

**Keywords:** Paediatrics, Public health

## Abstract

The dissociation of effects of age, time of assessment and cohort is a well-known challenge in developmental science. We examined effects of time of assessment in the school year on children’s physical fitness using data from 75,362 German third-graders from seven cohorts. Children were tested once either in the first or second school term of third grade. Tests examined cardiorespiratory endurance (6-min run), coordination (star-run), speed (20-m sprint), lower (standing long jump) and upper (ball-push test) limbs muscle power, and flexibility (stand-and-reach test). We estimated the effect of time of assessment using a regression discontinuity design specified in a linear mixed model with random factors child and school and adjusted for age, sex, and cohort effects. Coordination, speed, and upper limbs muscle power were better in second compared to first school term, with boys exhibiting a larger increase of upper limbs muscle power than girls. There was no evidence for changes in cardiorespiratory endurance, lower limbs muscle power, and flexibility between assessments. Previously reported age and sex effects as well as secular fitness trends were replicated. There is thus evidence for improvement of some physical fitness components beyond age and cohort effects that presumably reflects the benefit of physical activity in physical education and other settings. Effects of assessment time should be taken into consideration in performance-based grading or norm-based selection of children.

## Introduction

Physical fitness is an important health marker of children and adolescents^1–3^. Especially cardiorespiratory endurance is associated with better cardiovascular health and a lower risk for obesity^2,4^. Further, cardiorespiratory and muscular fitness are positively related to children’s health-related quality of life^5^, and cognitive function^6,7^.

There are well-known gender- and age-related effects on children’s physical fitness^8–10^. The direction of gender effects depends on the physical fitness component (also referred to as physical fitness dimension^11^). For example, boys outperform girls in cardiorespiratory endurance and muscle power but the reverse is true for static balance and flexibility^8,12,13^. The size of gender differences varies strongly by physical fitness component, for instance, gender differences are larger for muscle power than for coordination^8,14^. There is also age-related development of physical fitness^8–10^. Previous reports showed that within the ninth year of life (i.e., in third grade of primary school), age-related development is linear for six tests assessing cardiorespiratory endurance, coordination, speed, lower and upper limbs muscle power, and static balance, and developmental rates do not differ significantly between boys and girls. However, physical fitness components differ strongly in how much they develop within one year. For example, age effects in third grade are comparatively small for cardiorespiratory endurance and large for upper limbs muscle power^8,13,14^.

Besides these age-related physical fitness differences, physical fitness may differ by time of assessment. For instance, children tested in the second half of the school year may exhibit better physical fitness compared with children tested in the first half of the school year, due to physical activity accumulated in physical education and other settings. However, given that children’s performance in most fitness tests improves with increasing age, and children grow older throughout the school year, the question is whether the developmental effect related to the amount of physical activity accumulated over the school year can be dissociated from the confounded ontogenetic age-related developmental effect.

Time of assessment in the year has been the topic of some earlier research. In a study assessing the effects of a fitness intervention that was only conducted during the school year, children’s cardiorespiratory fitness improved throughout the school year, but declined to control group levels during the summer break^15^. Similar results were found in another study, where primary school children’s cardiorespiratory endurance was lower after, compared to before a 12-week summer break^16^. This loss of physical fitness during the summer illustrates the importance of sports opportunities like physical education classes and other school sports for fitness development, especially in children for whom school sports is the main source of moderate-to-vigorous activity. In these studies, a lack-of-exposure effect was counter to or stronger than the expected age-related improvement. Further, children’s body mass index may fluctuate during the year, and fitness declines may also be associated with weight gains during summer break^17,18^. In contrast, Drenowatz et al.^19^ reported better performance in the beginning than at the end of the school year in some fitness tests, and more monthly development in several fitness components during the summer break compared to the school year. In their study, 214 primary school children were tested at the beginning and end of each school year for a period of four years. Age- and sex-standardized scores in the 6-min run, push-ups, sit-ups, and standing long jump (i.e., cardiorespiratory endurance, muscular endurance, lower limbs muscle power) were higher at the beginning than at the end of the school year. They suggested that their results may be related to a summer-related increase of physical activity in their sample. However, performance in the 20-m sprint, balancing and jumping sideways was better at the end of the school year, while performance in the stand-and-reach test was not affected by time of assessment.

Finally, Hjorth et al. tested children’s physical fitness three times within a school year and reported higher cardiorespiratory endurance of third- and fourth-graders in spring, relative to the previous fall or winter^20^. However, this study did not adjust for age when estimating the effect of assessment time on physical fitness, making it uncertain whether fitness improvements were only due to differences in physical activity associated with different assessment times, or due to age-related development. In summary, previous research on the effects of time of assessment on children’s physical fitness reported a varied profile of results.

To test associations between children’s physical fitness and time of assessment within the school year, the present study used data from seven cohorts of third-graders, who were either tested in the *first* or in the *second school term of third grade*. Data from children tested in the *first* half of the school year were previously published in Fühner et al.^8^, who examined cross-sectional age-related fitness differences. Adding data of children from two previous cohorts who were tested in the *second*, instead of the *first* half of the school year allowed us (1) to test effects of time of assessment on physical fitness, dissociating them from previously reported age effects^8^, (2) to include data from an additional fitness test assessing flexibility which had not been published in the previous study, and (3) to test whether sex and age effects reported by Fühner et al.^8^ are moderated by time of assessment (i.e., age × school term and sex × school term interactions).

Due to the positive effects structured exercise may have on physical fitness^15,16^, and as children tested in the second school term have accumulated on average an additional half year of exercise in physical education classes and other settings like organized sports than children tested earlier in the school year, we expected better fitness of third-graders in the second compared to the first school term after statistically adjusting for age-related and cohort-related correlates.

We expected to replicate age and sex effects previously reported for five of the six tested fitness components^8^. As data used in the present study spans seven different cohorts (i.e., cohorts 2009–2015), we further expected to replicate cohort trends, specifically a decline of cardiorespiratory endurance and an increase of speed over the years^8,21^.

## Methods

### Experimental approach

Starting in school year 2009/10, the EMOTIKON research project (uni-potsdam.de/en/emotikon/) has annually tested the physical fitness of all third-graders in the Federal State of Brandenburg, Germany. EMOTIKON was mandated and approved by the Ministry of Education, Youth and Sport of Brandenburg. Based on the Brandenburg School Law, participation is obligatory for all public primary schools^22^.

The present study used data from cohorts 2009 until 2015. In the German school system, each school year has two school terms. The first school term usually begins in late summer or early fall (i.e., August or September) and the second school terms begins in winter, typically around mid-February, and lasts until the summer break (i.e., typically between end of June and mid-July). School-summer holidays last six weeks in Germany. In the school years 2009/10 and 2010/2011, fitness tests were conducted during the *second school term*, between April and June 2010 (i.e., cohort 2009) or mid-February to April 2011 (i.e., cohort 2010). Note that, because third-graders belonging to cohorts 2009 and 2010 were tested in their second school term, their data was collected in 2010 and 2011, respectively. Starting in school year 2011/12 (i.e., cohorts 2011 until 2015), the fitness tests were conducted in the *first school term* of third grade, between September and November.

Prior to the EMOTIKON tests, schools and parents received written information about the EMOTIKON research project, information on data processing and data protection. Schools received instructions on test administration. Research was conducted in accordance with the latest Declaration of Helsinki^23^ and the Brandenburg School Law^22^. The authors received the data completely anonymized from the Ministry of Education, Youth and Sport of the Federal State of Brandenburg. None of the researchers had access to personally identifiable information of the children.

### Participants

96,956 children participated in the EMOTIKON research project between the school years 2009/10 and 2015/16. Based on previous research that showed a delayed physical fitness development for children with delayed school enrollment^24,25^, we focused analyses on children with school enrollment according to the legal key date (i.e., keyage children). Analyses including children with delayed school enrollment (i.e., older-than-keyage children) are provided in the OSF repository. In their year of school enrollment, keyage children included in our main analyses had turned six before the legal key date, which is September 30 in the Federal State of Brandenburg, Germany. This left us with 75,398 children. We excluded children with a physical disability or autism (*N* = 35). For each physical fitness test and separately for boys and girls, test scores outside of a ± 3 *SD* range were excluded. This left us with 440,139 test scores from 75,362 children in 469 schools from seven cohorts. Table 1 provides a sample description including number of children and schools as well as children’s ages. For a more detailed sample description including children’s mean test scores in the first and second school term, see Table S1 in the Supplements.

**Table 1:** Sample description.

Time of assessment	N children	N schools	N observations	Age mean (SD)
1st school term (cohorts 2011 to 2015)	54,190 (50.6% girls)	462	317,565	8.51 (0.29)
2nd school term (cohorts 2009 + 2010)	21,172 (50.9% girls)	417	122,574	9.07 (0.31)
Total	75,362 (50.7% girls)	469	440,139	8.66 (0.39)

### Physical fitness tests

The EMOTIKON tests assess the six physical fitness components cardiorespiratory endurance (i.e., 6-min run), coordination (i.e., star-run), speed (i.e., 20-m linear sprint), lower (i.e., standing long jump) and upper (i.e., ball-push test) limbs muscle power, and flexibility (i.e., stand-and-reach test). Physical education teachers conducted the physical fitness tests, following a standard procedure (for more details, please see www.uni-potsdam.de/emotikon/projekt/methodik). Prior to the physical fitness tests, children received a warm-up consisting of running exercises and games. Children were encouraged to achieve their best performance in the physical fitness tests.

#### Cardiorespiratory endurance

The 6-min run assessed children’s cardiorespiratory endurance. For six minutes, children ran as far as possible around a field of the size 9 m × 18 m (≙ 54 m). The field was marked by six pylons that were set at a 9-m distance from each other. If a child stopped between two pylons at the stop signal, they were allowed to continue to the next pylon. The total running distance up to that last pylon was recorded in meters and used for analysis. In children aged 7 to 11 years, the 6-min run showed a test–retest reliability of *r* = 0.92^26^.

#### Coordination

The star-run was used to assess coordination under time pressure. Children had to run a star-like pattern with a total distance of 50.912 m as fast as possible. The pattern was marked by five pylons, four of which were set at the corners of a 9 m × 9 m field and one pylon marking the center. Starting from the center, children had to run to each of the other four pylons, touch it by hand and run back to the center. The order of movement directions and movement forms (i.e., running forward, running backward, side-steps to the right side, side-steps to the left side) that children had to use to complete the parkour was standardized. Time was measured with a 1/10 s accuracy. Each child completed two test trials of the star-run test, and the better result was used in analysis. In 8- to 10- year-old children, the star-run test showed a test–retest reliability (intra-class-correlation coefficient, ICC) of 0.68 (95% CI 0.53–0.79)^27^.

#### Speed

The 20-m linear sprint tested speed. Children stood in an upright position, one foot on the start line. After an acoustic start signal, they sprinted as fast as possible over a distance of 20 m. Time was measured in seconds with a 1/10 s accuracy. Children had two trials; the fastest trial was used for analysis. In children aged 7 to 11 years, the 20-m linear sprint test showed a test–retest reliability of *r* = 0.90^26^.

#### Lower limbs muscle power (PowerLOW)

The standing long jump tested muscle power of the lower limbs (PowerLOW). From a standing upright position with their feet parallel, children had to jump as far as possible. Children had to jump with both legs and land with both feet together; they were allowed to swing their arms before and during the jump but they were not allowed to touch the floor with their hands after landing. The jump distance between the starting line and their heels at landing was measured to the nearest centimeter. The children completed the standing long jump twice, the trial with the better jump distance was used for analysis. The standing long jump showed a test–retest reliability (ICC) of 0.94 (95% CI 0.93–0.95) in children aged 6 to 12 years^28^.

#### Upper limbs muscle power (PowerUP)

The ball-push test assessed muscle power of the upper limbs (PowerUP). Children had to hold a 1 kg medicine ball in front of their chest with their arms bent and then push the ball as far as possible with both hands. The pushing distance was measured in meters with a 10 cm accuracy. Each child completed the ball-push test twice. Again, the trial with the better pushing distance was used for analysis. In 8- to 10-year-old children, the ball push test showed a test–retest reliability (ICC) of 0.81 (95% CI 0.71–0.87)^27^.

#### Flexibility

Children’s flexibility was tested using the stand-and-reach test. Children stood barefoot with their feet together on a box on which a centimeter scale was attached. 100 cm marked the edge of the box. They stretched out their arms and held them shoulder-wide above their head. On an exhale, children bent their upper body forward, their knees remaining straight. With their fingertips, they reached down as far as possible on the centimeter scale. The final position was held for two seconds. Distance reached on the scale was measured to the nearest one centimeter. Distances above 100 cm indicated that children were able to reach beyond their toes, distances below 100 cm indicated that children did not reach their toes. Children had two trials; the better result was used for analysis. In children aged 7 to 11 years, the stand-and-reach test showed a test–retest reliability of *r* = 0.94^26^.

### Statistics

As data in the present study was collected in seven different cohorts, an effect of time of assessment must be dissociated from potential cohort-related changes in performance that vary in direction and size between physical fitness components^8,21^. This confound between cohort and time of assessment was addressed with a regression discontinuity design (RDD)^29,30^, which tested whether the change in time of assessment between cohorts 2010 and 2011 becomes visible in a step-up or step-down change in performance when extrapolating secular trends forward from 2009–2010 and backwards from 2015 to 2011 to the date of this design discontinuity (i.e., 2010.5). The assessment effect is thus computed as the difference between the intercept of the forwardly extrapolated secular trend of 2009–2010 cohorts and the intercept of the backwardly extrapolated secular trend of 2015–2011 cohorts. The RDD allowed for a change in linear secular trends before and after the change in assessment time (i.e., before and after 2010.5).

Data preprocessing and analysis was done using *R* (4.2.3)^31^, the *RStudio IDE*^32^, and *Julia* (Version 1.9)^33^. For data preprocessing, we used the *tidyverse* packages^34^ in *R*. Linear mixed models (LMMs) were fit using the *MixedModels.jl*^35^ and *MixedModelsMakie.jl*^36^ packages in *Julia*. Partial effects were computed with the *MixedModelExtras.jl* package^37^ in *Julia*.

Preprocessing was similar to the one reported in previous studies^8,13^. Based on a Box-Cox distributional analysis^38^, a reciprocal transformation of the star-run and the 20-m sprint scores brought model residuals in line with a normal distribution. For the stand-and-reach test, test scores were squared.

The original unit of the star-run and the 20-m sprint test was seconds. We transformed their units into meters/seconds by multiplying the reciprocal scores (1/seconds) of the star-run with 50.912 (distance in meters of the star-run) and the reciprocal scores of the 20-m sprint with 20 (distance in meters of the 20-m sprint). Consequently, for all six fitness tests, higher scores indicate a better performance.

We first calculated z-scores for each physical fitness test, separately for boys and girls. Scores outside of a ± 3 *SD* range were defined as outliers and excluded from data analysis. We then recalculated z-scores for each physical fitness test aggregated over boys and girls, to keep gender-related differences in the data.

Contrasts were similar to the ones specified in previous analyses^8,13,14^. For the six-level factor physical fitness component, we specified five contrasts comparing (1) cardiorespiratory **e**ndurance versus **c**oordination, **s**peed and power**L**OW (i.e., cardiorespiratory endurance versus tests of acceleration, E versus CSL), (2) **c**oordination versus **s**peed and power**L**OW (C versus SL), (3) **s**peed versus power**L**OW (S versus L), (4) cardiorespiratory **e**ndurance, **c**oordination, **s**peed and power**L**OW versus power**U**P (ECSL versus U), and (5) cardiorespiratory **e**ndurance, **c**oordination, **s**peed and power**L**OW versus **f**lexibility (ECSL versus F). These contrasts were motivated by the fact that the first four fitness components are positively correlated and indicative of the latent construct “physical fitness”, whereas correlations between powerUP and the other fitness components are lower^8,13,14^. Similarly, as flexibility is neither energetically determined nor information-oriented, but reflects a passive system of energy transmission^39^, correlations between flexibility (F) and the first four physical fitness components (ECSL) were also expected to be low. Moreover, expected sex differences in flexibility are qualitatively different from the other tests (i.e., girls > boys)^10,12^. The factor assessment was dummy coded, with “first school semester” as reference category. A sequential difference contrast of the factor sex compared the physical fitness of boys and girls, with positive estimates indicating a better performance of boys, and negative estimates indicating a better performance of girls. Age was centered at 8.5 years, and cohort was centered at 2010.5.

Parsimonious RDD-based LMM selection^40^ is documented in script *Assessment.qmd* in the OSF repository (https://osf.io/4vj2q/). The goal was to fit an LMM that included all relevant variance components and correlation parameters without overparameterization. We started with a complex LMM including the fixed effects of assessment, sex, a second-order polynomial age trend, a third-order polynomial cohort trend and interactions between fixed effects, all nested under the factor levels of physical fitness component. A quadratic age and a cubic cohort effect and interactions between sex × age or sex × cohort indicated overparameterization and were dropped. The final LMM included the fixed effects for assessment, sex, age (linear), cohort (linear and quadratic), the interaction between assessment and sex, the interaction between assessment and age, and the interaction between assessment and cohort (linear), all nested under the levels of the factor physical fitness component. A significant positive interaction between assessment and the linear cohort trend indicates that the linear slope across cohorts with assessment in the second school term (i.e., cohorts 2009 and 2010) was larger than the linear slope for cohorts with assessment in the first school term (i.e., cohorts 2011–2015).

For the random factor school (*N* = 469), we included variance components of physical fitness component, sex, age, and the interaction between assessment and cohort (linear), with assessment and cohort as well as their interaction nested under the levels of physical fitness component. Correlation parameters were included for all variance components except for sex. For the random factor child (*N* = 75,362), we included physical fitness component-related variance components and correlation parameters. In line with earlier practice, we interpreted effects with|z| ≥ 2.0 as statistically significant.

### Ethical approval and consent to participate

The EMOTIKON project is mandated and approved by the Ministry of Education, Youth and Sport of the Federal State of Brandenburg, Germany. According to the Brandenburg School Law, participation is mandatory for all public primary schools in the Federal State of Brandenburg, Germany^22^. Written consent to participate is not required. Research was conducted in accordance with the latest Declaration of Helsinki^23^ and the Brandenburg School Law^22^.

## Results

**Figure 1:**
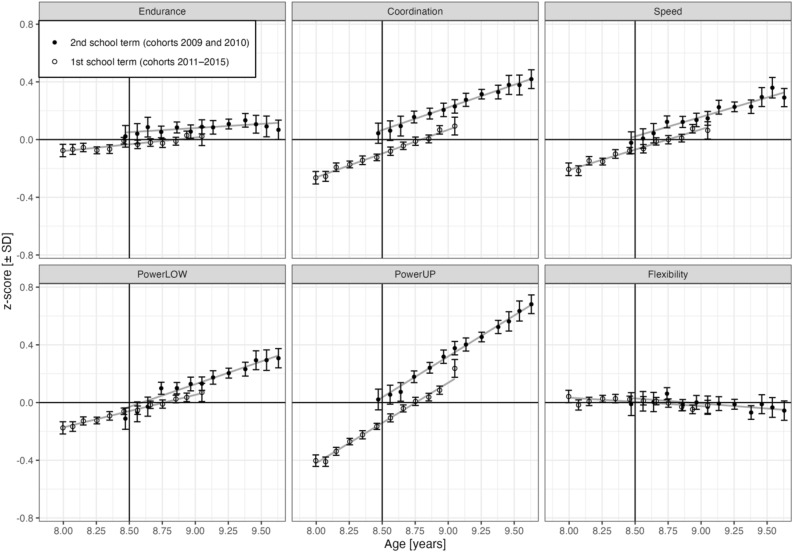
Physical fitness by age and time of assessment (first versus second school term). Points are binned child means with 95% CIs. Endurance = cardiorespiratory endurance (i.e., 6-min run), Coordination = star-run, Speed = 20-m linear sprint, PowerLOW = lower limbs muscle power (i.e., standing long jump), PowerUP = upper limbs muscle power (i.e., ball-push test), Flexibility = stand-and-reach test. Age was centered at 8.5 years (indicated by vertical line).

**Figure 2:**
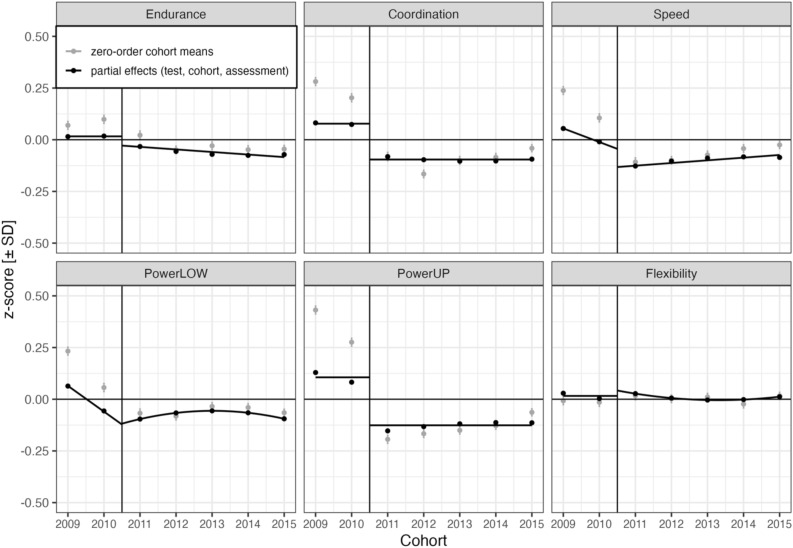
Physical fitness by cohort and time of assessment. The vertical line at 2010.5 separates cohorts with assessment in second school term (i.e., 2009 and 2010) from cohorts with assessment in first school term (i.e., cohorts 2011–2015). Grey points show zero-order cohort means with 95% CIs. Black points and lines show partial effect predictions with effects of physical fitness test, cohort, and assessment. Differences between black partial effect predictions and grey zero-order means are due to statistical adjustments for age and sex, as well as due to individual differences between children in physical fitness and individual differences between schools in physical fitness, assessment, age, sex, and cohort effects. Endurance = cardiorespiratory endurance (i.e., 6-min run), Coordination = star-run, Speed = 20-m linear sprint, PowerLOW = lower limbs muscle power (i.e., standing long jump), PowerUP = upper limbs muscle power (i.e., ball-push test), Flexibility = stand-and-reach test.

The profile of results is visualized in Fig. 1, displaying physical fitness for the six fitness components by age and time of assessment (i.e., first versus second school term). A table of corresponding means and standard deviations in the original task metrics is available as Table S1 in the Supplement. LMM-based inferential fixed effect estimates and associated standard errors and z-values are assembled in Table 2; variance components and correlation parameters related to the random effects child and school are shown in Table 3. Figure 2, finally, provides visualizations of partial effect predictions based on LMM parameters with a focus on RDD effects. In the following, we report results for each of the six physical fitness components with reference to Fig. 1, Table 2, and Fig. 2.

**Table 2:** Fixed effect estimates, standard errors and z-values of the linear mixed model.

	*b*	SE	z
Intercept	− 0.079	0.016	− **5.07**
E versus CSL	0.093	0.029	**3.18**
C versus SL	0.059	0.038	1.56
S versus L	− 0.024	0.032	− 0.75
ECSL versus U	0.079	0.028	**2.83**
ECSL versus F	− 0.130	0.026	− **5.08**
*Cardiorespiratory endurance (6-min run)*			
Assessment	0.040	0.041	0.97
Sex	0.514	0.008	**61.50**
Assessment × sex	− 0.007	0.015	− 0.49
Age 2011–2015 (linear)	0.097	0.014	**6.85**
Δ Age 2009–2010 (linear)	− 0.036	0.027	− 1.33
Cohort 2011–2015 (linear)	− 0.033	0.015	− **2.15**
Δ Cohort 2009–2010 (linear)	0.044	0.038	1.17
Cohort 2009–2015 (quadratic)	0.005	0.003	1.81
*Coordination (star-run)*			
Assessment	0.144	0.046	**3.17**
Sex	0.237	0.008	**28.62**
Assessment × sex	0.005	0.015	0.35
Age 2011–2015 (linear)	0.317	0.014	**22.56**
Δ Age 2009–2010 (linear)	− 0.021	0.026	− 0.79
Cohort 2011–2015 (linear)	− 0.023	0.016	− 1.41
Δ Cohort 2009–2010 (linear)	0.023	0.043	0.55
Cohort 2009–2015 (quadratic)	0.004	0.003	1.57
*Speed (20-m sprint)*			
Assessment	0.097	0.046	**2.10**
Sex	0.307	0.008	**36.75**
Assessment × sex	− 0.013	0.015	− 0.90
Age 2011–2015 (linear)	0.262	0.014	**18.52**
Δ Age 2009–2010 (linear)	− 0.050	0.027	− 1.90
Cohort 2011–2015 (linear)	0.032	0.016	**2.09**
Δ Cohort 2009–2010 (linear)	− 0.105	0.040	− **2.66**
Cohort 2009–2015 (quadratic)	− 0.004	0.003	− 1.74
*PowerLOW (standing long jump)*			
Assessment	− 0.006	0.037	− 0.16
Sex	0.372	0.009	**43.30**
Assess × sex	0.010	0.015	0.68
Age 2011–2015 (linear)	0.227	0.015	**15.59**
Δ Age 2009–2010 (linear)	− 0.003	0.027	− 0.10
Cohort 2011–2015 (linear)	0.049	0.015	**3.35**
Δ Cohort 2009–2010 (linear)	− 0.189	0.032	− **5.87**
Cohort 2009–2015 (quadratic)	− 0.010	0.003	− **3.78**
*PowerUP (ball-push test)*			
Assessment	0.222	0.037	**5.98**
Sex	0.645	0.008	**81.12**
Assessment × sex	0.053	0.014	**3.76**
Age 2011–2015 (linear)	0.519	0.013	**38.78**
Δ Age 2009–2010 (linear)	− 0.031	0.025	− 1.23
Cohort 2011–2015 (linear)	0.028	0.014	1.97
Δ Cohort 2009–2010 (linear)	− 0.082	0.035	− **2.33**
Cohort 2009–2015 (quadratic)	− 0.004	0.002	− 1.51
*Flexibility (stand-and-reach test)*			
Assessment	− 0.048	0.033	− 1.43
Sex	− 0.429	0.009	− **49.20**
Assessment × sex	− 0.069	0.016	− **4.41**
Age 2011–2015 (linear)	− 0.045	0.015	− **3.03**
Δ Age 2009–2010 (linear)	− 0.004	0.028	− 0.14
Cohort 2011–2015 (linear)	− 0.033	0.015	− **2.27**
Δ Cohort 2009–2010 (linear)	0.019	0.031	0.61
Cohort 2009–2015 (quadratic)	0.006	0.003	**2.25**

Δ Cohort/Age 2009–2010 (linear) = change in linear slope from Cohort/Age 2011–2015. Endurance = cardiorespiratory endurance (i.e., 6-min run), coordination = star-run, Speed = 20-m linear sprint, PowerLOW = lower limbs muscle power (i.e., standing long jump), PowerUP = upper limbs muscle power (i.e., ball-push test), Flexibility = stand-and-reach test. E versus CSL = cardiorespiratory endurance versus coordination, speed and powerLOW, C versus SL = coordination versus speed and powerLOW, S versus L = speed versus powerLOW, ECSL versus U = cardiorespiratory endurance, coordination, speed and powerLOW versus powerUP, ECSL versus F = cardiorespiratory endurance, coordination, speed and powerLOW versus flexibility.

Bold =|z|> 2.0, linear mixed model random factors: schools (469) and children (75,362), observations = 440,139. For estimates of variance components and correlation parameters, see Table 3.

**Table 3:** Child- and school-related variance components and correlation parameters of the linear mixed model.

	VC	CP											
		Int	E_CSL	C_SL	S_L	ECSL_U	ECSL_F	Assessment					
								E	C	S	pL	pU	
Child													
Int	0.315												
E_CSL	0.407	− 0.15											
C_SL	0.370	− 0.09	+ 0.01										
S_L	0.363	− 0.09	+ 0.07	+ 0.05									
ECSL_U	0.530	**+ 0.25**	+ 0.16	− 0.07	+ 0.06								
ECSL_F	0.793	**+ 0.21**	+ 0.02	− 0.05	+ 0.11	**+ 0.26**							
School													
Int	0.061												
E_CSL	0.273	− 0.03											
C_SL	0.519	**+ 0.24**	− 0.15										
S_L	0.337	**+ 0.23**	− 0.08	− 0.12									
ECSL_U	0.229	**+ 0.25**	− 0.04	+ 0.21	+ 0.21								
ECSL_F	0.149	**+ 0.49**	+ 0.02	+ 0.25	+ 0.12	+ 0.42							
Assessment													
E	0.433	− 0.22	− **0.34**	− 0.02	+ 0.02	+ 0.01	− 0.16						
C	0.576	− 0.20	+ 0.23	− **0.36**	+ 0.03	− 0.11	− 0.14	− 0.08					
S	0.606	− 0.17	+ 0.13	+ 0.15	− **0.37**	− 0.21	− 0.11	+ 0.04	+ 0.19				
pL	0.320	− 0.07	+ 0.09	+ 0.23	**+ 0.34**	+ 0.12	− 0.11	+ 0.10	+ 0.16	+ 0.15			
pU	0.348	− 0.10	+ 0.02	+ 0.16	+ 0.03	**+ 0.41**	+ 0.00	+ 0.01	+ 0.00	+ 0.01	+ 0.25		
F	0.219	− 0.03	− 0.08	+ 0.08	− 0.12	+ 0.10	**+ 0.42**	+ 0.10	− 0.11	+ 0.04	− 0.20	+ 0.08	
Cohort 2011–2015 (linear)													
E	0.020	− **0.33**	− **0.64**	− 0.06	− 0.06	− 0.24	− 0.31	**+ 0.41**	− 0.07	− 0.01	− 0.04	− 0.12	
C	0.035	− **0.48**	+ 0.29	− **0.72**	− 0.01	− 0.27	− 0.39	+ 0.06	**+ 0.45**	+ 0.14	+ 0.01	− 0.08	
S	0.025	− **0.36**	+ 0.23	+ 0.29	− **0.63**	− 0.22	− 0.20	+ 0.03	+ 0.04	**+ 0.53**	+ 0.05	+ 0.05	
pL	0.011	− 0.19	+ 0.21	+ 0.26	**+ 0.40**	− 0.04	− 0.14	+ 0.08	+ 0.04	+ 0.10	**+ 0.55**	+ 0.13	
pU	0.016	− **0.30**	− 0.07	+ 0.04	− 0.04	**+ 0.62**	− 0.05	+ 0.12	− 0.01	− 0.06	+ 0.12	**+ 0.44**	
F	0.009	− 0.16	+ 0.05	+ 0.02	− 0.19	− 0.02	**+ 0.50**	+ 0.02	− 0.02	+ 0.16	− 0.10	− 0.04	
Δ Cohort 2009–2010 (linear)													
E	0.319	+ 0.13	+ 0.25	+ 0.07	+ 0.07	+ 0.21	+ 0.12	+ 0.57	− 0.03	− 0.01	+ 0.12	+ 0.01	
C	0.467	+ 0.27	− 0.06	+ 0.44	− 0.00	+ 0.18	+ 0.29	− 0.16	+ 0.44	+ 0.05	+ 0.21	+ 0.06	
S	0.368	+ 0.12	− 0.04	− 0.09	+ 0.19	− 0.03	+ 0.09	− 0.03	+ 0.15	+ 0.58	+ 0.11	− 0.08	
pL	0.164	+ 0.21	− 0.11	+ 0.06	+ 0.03	+ 0.16	+ 0.09	+ 0.07	+ 0.08	+ 0.04	+ 0.60	+ 0.17	
pU	0.272	+ 0.23	+ 0.14	+ 0.12	+ 0.07	− 0.14	+ 0.06	− 0.17	+ 0.07	+ 0.06	+ 0.04	+ 0.54	
F	0.139	+ 0.17	− 0.12	+ 0.03	− 0.01	+ 0.12	+ 0.07	+ 0.08	− 0.19	− 0.06	− 0.17	+ 0.11	
Age	0.003	**+ 0.33**	+ 0.24	− 0.09	+ 0.28	+ 0.07	+ 0.23	− 0.05	+ 0.24	+ 0.00	+ 0.20	− 0.30	
Sex	0.003	–	–	–	–	–	–	–	–				

	CP												
	Assessment	Cohort 2011–2015 (linear)						Δ Cohort 2009–2010 (linear)					
	F	E	C	S	pL	pU	F	E	C	S	pL	pU	F
Cohort 2011–2015 (linear)													
E	+ 0.03												
C	− 0.07	+ 0.10											
S	+ 0.08	+ 0.05	+ 0.08										
pL	− 0.06	− 0.02	+ 0.05	+ 0.11									
pU	+ 0.11	+ 0.06	+ 0.08	+ 0.15	+ 0.07								
F	**+ 0.46**	+ 0.01	+ 0.08	+ 0.22	− 0.01	+ 0.04							
Δ Cohort 2009–2010 (linear)													
E	+ 0.01	− 0.33	− 0.07	− 0.03	+ 0.13	+ 0.03	− 0.03						
C	− 0.08	− 0.17	− 0.43	+ 0.01	+ 0.01	− 0.09	− 0.04	+ 0.01					
S	+ 0.01	− 0.01	+ 0.11	− 0.20	+ 0.08	− 0.16	+ 0.06	− 0.04	+ 0.04				
pL	− 0.13	− 0.02	− 0.10	− 0.06	− 0.15	− 0.00	− 0.05	+ 0.09	+ 0.23	+ 0.07			
pU	+ 0.03	− 0.21	− 0.17	− 0.05	+ 0.09	− 0.31	− 0.05	+ 0.02	+ 0.19	+ 0.07	+ 0.11		
F	+ 0.67	+ 0.03	− 0.11	− 0.09	− 0.10	+ 0.06	− 0.14	+ 0.03	− 0.15	+ 0.03	− 0.04	+ 0.13	
Age	− 0.09	+ 0.03	+ 0.18	− 0.06	+ 0.26	− 0.06	+ 0.24	+ 0.09	+ 0.09	+ 0.33	− 0.02	− 0.12	− 0.23
Sex													

E = cardiorespiratory endurance (i.e., 6-min run), C = coordination (i.e., star-run), S = speed (i.e., 20-m linear sprint), pL = lower limbs muscle power (i.e., powerLOW, standing long jump), pU = upper limbs muscle power (i.e., powerUP, ball-push test), F = flexibility (i.e., stand-and-reach test). E_CSL = cardiorespiratory endurance versus coordination, speed and powerLOW, C_SL = coordination versus speed and powerLOW, S_L = speed versus powerLOW, ECSL_U = cardiorespiratory endurance, coordination, speed and powerLOW versus powerUP, ECSL_F = cardiorespiratory endurance, coordination, speed and powerLOW versus flexibility. Assessment = Assessment effect estimated at 2010.5, Cohort 2011–2015 (linear) = Linear cohort trend between 2011 and 2015, Δ Cohort 2009 – 2010 (linear) = Change in linear cohort slope from linear cohort trend between 2011 and 2015. VC = variance component, CP = correlation parameter.

Theoretically relevant correlations are set in bold.

LMM random factors: schools (469) and children (75,362), observations = 440,139. VC for Residual = 0.192.

### Assessment and cohort effects

#### Cardiorespiratory endurance

There was no evidence for a better 6-min run performance in the second compared to the first school term (i.e., no significant assessment effect in 2010.5). As expected and reported previously^8,21^, there was a small but significant decline in the 6-min run performance (*b* = − 0.033, z = − 2.15, see Fig. 2), with no evidence for an interaction of the cohort trend with assessment.

#### Coordination

As shown in Fig. 1 for age and in Fig. 2 for cohort, children exhibited better star-run performance when they were tested in the second compared to the first school term (*b* = 0.144, z = 3.17). The graph for physical fitness by cohort (Fig. 2) shows a discontinuity of cohort trends at 2010.5 (i.e., between cohorts with assessment in first and second school term), indicating an assessment effect on test performance. Grey points are observed cohort means, black points are partial effects of physical fitness test, cohort, and assessment (i.e., without effects of age or sex). As fixed effect estimates describe changes in units of standard deviation, b*SD translates these effects into their original test metric. For the star-run, the positive assessment effect translates to a performance increase of 0.042 m/s from first to second school term. As shown in Fig. 2, there was no evidence for linear or quadratic cohort trends of the star-run test performance.

#### Speed

Children tested in the second school term outperformed children tested in the first school term in the 20-m sprint (*b* = 0.097, z = 2.10), which translates to a performance difference of 0.04 m/s. Again, this effect is visible in the discontinuity of cohort trends at 2010.5 shown in Fig. 2. As expected, speed increased linearly in cohorts 2011–2015 (*b* = 0.032, z = 2.09), but the cohort effect differed between cohorts with assessment in second (2009 + 2010) and first (2011–2015) school term (*b* = − 0.105, z = − 2.66). A re-parameterized LMM with the linear cohort trend nested under the levels of assessment showed that 20-m sprint performance declined from 2009 to 2010 (*b* = − 0.073, z = − 2.04). Details on this LMM are reported in script *Assessment.qmd* in the OSF repository.

#### PowerLOW

As shown in Fig. 1 for age and in Fig. 2 for cohort, there was no evidence for a change of performance in the standing long jump between first and second school semester estimated at 2010.5. Standing long jump performance was characterized by a linear increase (*b* = 0.049, z = 3.35) during cohorts 2011–2015 and an overall quadratic decline (*b* = − 0.010, z = − 3.78). Linear cohort trends differed between assessments (*b* = − 0.189, z = − 5.87). A re-parameterized LMM with the linear cohort trend nested under the levels of assessment (i.e., first and second school term) showed a linear decline of standing long jump performance before 2010.5 (*b* = − 0.140, z = − 5.28).

#### PowerUP

Children’s performance in the ball-push test was better in the second compared to the first school term (*b* = 0.222, z = 5.98), which translates to a performance difference of 17 cm when estimated at 2010.5. Linear and quadratic cohort trends were not significant, but there was an interaction between the linear cohort effect and assessment time (*b* = − 0.082, z = − 2.33). In a re-parameterized LMM with the linear cohort trend nested under the levels of assessment (i.e., first and second school semester), neither cohort trend was significant, but there was a nonsignificant decreasing trend for cohorts 2009 and 2010 (*b* = − 0.055, z = − 1.86) and a nonsignificant increasing trend between 2011 and 2015 (*b* = 0.027, z = 1.93).

#### Flexibility

There was no evidence for a significant main effect of assessment on stand-and-reach performance. As shown in Fig. 2, there was a small linear decline of the stand-and-reach test performance between 2011 and 2015 (*b* = − 0.033, z = − 2.27), followed by a plateau (*b* = 0.006, z = 2.25). There was no evidence for an interaction between linear cohort trend and assessment.

### Age and sex effects and interactions with assessment

As reported in previous studies^8,14^ and shown in Fig. 1, performance increased linearly with age in cohorts 2011–2015 for the five physical fitness components cardiorespiratory endurance (*b* = 0.097, z = 6.85), coordination (*b* = 0.317, z = 22.56), speed (*b* = 0.262, z = 18.52), powerLOW (*b* = 0.227, z = 15.59), and powerUP (*b* = 0.519, z = 38.78). Going beyond earlier results, there was no evidence that age-related development in these physical fitness components differed between first and second school term. Interestingly, flexibility was the only physical fitness component with a small negative age effect (*b* = − 0.045, z = − 3.03); this age effect also did not differ significantly between assessments. Boys outperformed girls in five of six physical fitness tests assessing cardiorespiratory endurance (*b* = 0.514, z = 61.50), coordination (*b* = 0.237, z = 28.62), speed (*b* = 0.307, z = 36.75), powerLOW (*b* = 0.372, z = 43.30), and powerUP (*b* = 0.645, z = 81.12). For the first four physical fitness components, there was no evidence that sex effects differed between assessments. For powerUP, however, there was a significant assessment × sex interaction (*b* = 0.053, z = 3.76), indicating that boys’ performance improved more than girls’ from first to second school semester. Flexibility was the only physical fitness component where girls outperformed boys (*b* = − 0.429, z = − 49.20, see Figure S1 in the Supplements). There was a significant assessment × sex interaction for flexibility (*b* = − 0.069, z = − 4.41), indicating that the girls’ performance advantage was slightly larger in the second compared to the first school term.

### Differences between physical fitness components in their assessment effects

Are physical fitness components differently affected by time of assessment? A re-parameterized version of the LMM estimated the interactions of the physical fitness component *contrasts* with assessment time. Details on this re-parameterized LMM are reported in the OSF repository. The performance increase from first to second school term was (1) larger for speed than for powerLOW (S versus L, *b* = 0.105, z = 2.03), (2) larger for powerUP than for the mean of cardiorespiratory endurance, coordination, speed, and powerLOW (ECSL versus U, *b* = − 0.151, z = − 3.68), and (3) larger for the mean of cardiorespiratory endurance, coordination, speed, and powerLOW than for flexibility (ECSL versus F, *b* = 0.115, z = 2.83). Differences in assessment effects between cardiorespiratory endurance and the mean of coordination, speed, and powerLOW (E versus CSL), or between coordination and the mean of speed and powerLOW (C versus SL) were not significant. There were two significant three-way interactions involving physical fitness contrasts, assessment, and sex (ECSL versus U  ×  assessment  ×  sex: *b* = − 0.054, z = − 3.72; ECSL versus F  ×  assessment  ×  sex: *b* = 0.068, z = 4.01). The first interaction indicates that for both, boys and girls, the performance gain from first to second school semester was larger for powerUP than for the mean of cardiorespiratory endurance, coordination, speed, and powerLOW (ECSL), but the difference in the performance gain between the fitness components was larger for boys. The second three-way interaction indicates that the difference between ECSL and flexibility in their development from first to second school term was more pronounced in boys compared to girls. While ECSL increased from first to second school term for both, boys and girls, flexibility (insignificantly) declined, and this decline was more pronounced (although not significant) for boys than for girls.

### Variance components (VCs) and correlation parameters (CPs)

Table 3 shows child- and school-related VCs and CPs. Conceptually, CPs represent interactions between the random factor and its associated VCs, or interactions between two effects when adjusting for all fixed effects.

#### Replication of previous findings and their extension by flexibility

Variance of physical fitness test contrasts were larger for children (i.e., VC range between 0.363 and 0.793) than for schools (i.e., VCs range between 0.149 and 0.519). CPs were in agreement with previous results indicating that the first four tests represent a well-defined latent construct of physical fitness, while powerUP is correlated much weaker with this cluster^8^. The newly added component flexibility was also weakly correlated with the first four physical fitness components. In a re-parameterized LMM with physical fitness *levels* instead of *contrasts* in the random effect structure, cardiorespiratory endurance, coordination, speed and powerLOW correlated with CPs between 0.57 and 0.77 on the child level, but correlations between powerUP and flexibility with the other four fitness components were lower (*r* between 0.24 and 0.49 for powerUP and between 0.21 and 0.34 for flexibility; powerUP and flexibility correlated at 0.20). Details on this LMM are reported in script *Assessment.qmd* in the OSF repository. As shown in Table 3, for both children and schools, the contrasts ECSL_U and ECSL_F correlated positively with the intercept (child: 0.25 and 0.21, school: 0.25 and 0.49, respectively), indicating that children and schools with higher average fitness estimated at 2010.5 showed better performance in ECSL (cardiorespiratory endurance, coordination, speed and powerLOW) than in powerUP or flexibility. Further, as reported previously^8^, the schools’ intercept and their age effect correlated positively (*r* = 0.33), indicating that fitter schools tended to exhibit larger cross-sectional age gains.

#### Differences between schools in their assessment effects and cohort trends

Schools differed in their assessment effects (i.e., VCs between 0.219 and 0.606) and in their linear cohort trends between 2011 and 2015 (i.e., VCs between 0.009 and 0.035). For both, assessment effects and cohort trends, CPs were in line with the “law of diminishing returns”^41,42^, indicating that schools with higher average performances in 2010.5 (1) exhibited smaller assessment effects and (2) were less likely to exhibit secular physical fitness gains between 2011 and 2015. Schools with lower average performances and possibly less active children may have had “more to gain” by increased amount of structured exercise and thus exhibited larger assessment effects. There were also positive CPs between assessment effects and the linear cohort trends between 2011 and 2015 of the corresponding physical fitness components (CPs between 0.41 and 0.55), indicating that schools promoting larger assessment effects were more likely to exhibit positive cohort trends between 2011 and 2015. Finally, schools also differed in the magnitude of change in linear cohort slopes before and after 2010.5 (i.e., Δ Cohort 2009–2010 [linear], VCs between 0.139 and 0.467). The CPs between the change in cohort slope and assessment or cohort effects must be interpreted with caution. We had no explicit prediction of their direction; they may arise from an assessment effect limiting the range of an associated cohort effect (or vice versa). Further details on CPs presented in Table 3 are documented in the Supplementary Material of this article.

## Discussion

We examined effects of time of assessment in the school year on children’s physical fitness using data from 75,362 German third-graders from seven cohorts. Children were tested either in the first or second school term of third grade in primary school. As time of assessment was confounded with age and cohort, we used a regression discontinuity design to dissociate assessment effects from linear age effects and quadratic cohort trends of physical fitness. Children’s coordination, speed, and upper limbs muscle power were higher in the second, compared to the first school term. Boys exhibited a larger improvement of upper limbs muscle power from first to second school term than girls. Upper limbs muscle power improved more from first to second school term than the mean of cardiorespiratory endurance, coordination, speed and lower limbs muscle power, four highly correlated physical fitness components. There was no reliable evidence for changes in cardiorespiratory endurance, powerLOW or flexibility from first to second school term.

The primary reason for better coordination, speed and upper limbs muscle power later in the school year while adjusting for children’s ages arguably is most likely that children in the second school term had accumulated on average an additional half year of physical activity in physical education classes, organized sports, or leisure time. The additional half year of physical education is the common denominator of children tested in the second half of the school year, as all primary school children are exposed to structured exercise in physical education classes, while not all children have access to sports clubs.

Improvements in some, but not all physical fitness components may be related to lesson content and physical activity intensity in physical education classes. In the Federal State of Brandenburg, Germany, primary school children usually receive three weekly physical education lessons with a duration of 45 min each^43^. Although activity levels vary between classes and children^44^, some studies have reported that primary school children spend on average less than half of physical education class time in moderate to vigorous physical activity^44–48^. Short bouts of intense physical activity as they occur in games of catch or ball games may promote speed, powerUP, and coordination, while improvements in cardiorespiratory endurance may require longer stimuli of moderate to vigorous intensity or multiple repetitions of short, high-intensity impulses^49^. Possibly, a higher prevalence of ball games in physical education relative to activities that might benefit standing long jumps may also explain the improvement for upper but not lower limbs muscle power from first to second school term.

In the present study, coordination was assessed by the star-run, in which children had to memorize different directions and forms of movement in a specific order. As the star-run is associated with a high cognitive load, an improvement from first to second school term might not only reflect better fitness, but also improved executive function.

In line with the improvements in coordination and speed from first to second school term, a previous study found better age- and sex-standardized performance of primary school children in tests assessing coordination and speed (i.e., 20-m sprint, backwards balancing, and jumping sideways) at the end of the school year, compared to the beginning. However, they reported better 6-min run, standing long jump, sit-up and push-up performance at the beginning of the school year after the summer holidays, suggesting an association with a summer-related increase of physical activity in their sample^19^.

Schools differed in their assessment effects, likely related to differences in schools’ physical education lessons. In line with the “law of diminishing returns”, results indicated that schools with a lower average fitness at 2010.5 tended to exhibit larger assessment effects. We did not expect this result, and we would have been able to explain the opposite effect by assuming that schools with a higher average fitness conduct more effective physical education classes and are located in areas with more opportunities to be physically active, and thus may also promote larger fitness gains (i.e., assessment effects) within the school year. Possibly, larger assessment effects in low fitness schools may be due to more pronounced fitness declines during the summer break in these schools, and a subsequent stronger fitness rebound over the school year, as children in these schools may have “more room” for positive development. According to the “structured day hypothesis”^17,50^, children tend to exhibit less favorable patterns in physical activity, sleep, and eating behavior on days without a consistent, formal structure, which are common during summer break, than on structured days like school days. However, it is likely that not all children experience unfavorable behavioral changes related to “unstructured” summer days^18,19^, and summer-related fitness declines may predominantly affect inactive children with a lack of access to sports programs during summer^15,16,18,51,52^.

Future research is needed to examine which specific factors are associated with larger effects of assessment time within the school year. As children in our study were tested in different seasons (i.e., fall in the first school term and winter or spring in the second school term), assessment-time related fitness differences may be associated with seasonal variations in physical activity or anthropometric measures. There is evidence for associations of children’s activity levels with seasonal variables, like temperature^53–55^, precipitation^53–55^ and hours of daylight^53–55^. Some research indicates that children tend to exhibit higher activity levels and less sedentary behavior in spring or summer, compared to fall or winter^20,56,57^, and there is evidence that children’s performance in several physical fitness tests is better in summer than in winter^58^. Moreover, in addition to seasonal differences in body composition^59^, some studies have even suggested seasonal variations in children’s height gains^59–61^.

Another factor likely related to differences in fitness gains over the course of the school year is the effectiveness of physical education lessons, that can differ depending on their quantity and quality (e.g., teaching strategies used and physical activity intensity)^62^. While in the Federal State of Brandenburg, Germany, children usually receive three physical education lessons per week, the distribution of the lesson quota on different school semesters and school grades can differ between schools^43^. Further, in cases of teacher shortage, the number of physical education lessons per week might be temporarily reduced in some schools and schools can thus differ in their exact amount of physical education classes. We do not have this information, but future studies may take into account school-specific amount and content of physical education lessons, teaching strategies^62^, or time spent in moderate-to-vigorous activity during physical education class^63,64^ when examining effects of time of assessment on children’s physical fitness. Besides physical education, children can acquire physical activity in sports clubs or leisure activities. As sports club participation is associated with better physical fitness^65^, and as children in sports clubs likely accumulate more structured exercise throughout the school year compared to children without access to organized sports, future reports may test whether assessment effects are moderated by sports club participation.

Besides effects on assessment time on physical fitness, the present study tested effects of age and sex on children’s fitness levels. Data from cohorts 2011 until 2015 including five fitness tests (i.e., assessing cardiorespiratory endurance, coordination, speed, powerLOW and powerUP) have been analyzed and published previously^8^, and we expected to replicate age and sex differences in these fitness tests. As expected, boys outperformed girls in five fitness tests assessing cardiorespiratory endurance, coordination, speed, and muscle power. The better performance of boys in these tests is likely related to differences in body composition^66,67^, endocrine levels^68^, and activity levels^20,55,69,70^. Pre-adolescent boys tend to exhibit lower fat and higher lean mass than pre-adolescent girls^66,67^. In fact, recent analyses have shown that after statistically adjusting for differences in body constitution (i.e., height-mass ratio), partial effects of sex on physical fitness no longer favored boys^14^. There is also evidence that school-aged boys tend to exhibit higher activity levels than girls^20,55,69,70^, indicated by higher daily step counts^70^, more time in moderate-to-vigorous physical activity^20,55,70^ and less sedentary behavior^55,69^.

Our study included data from an additional sixth fitness test, namely the stand-and-reach test, assessing flexibility. In line with previous research^10,12,58,71^, girls exhibited better flexibility than boys. In contrast to the other fitness components tested in the present study, performance in the stand-and-reach test does not depend on energetically determined or information-oriented abilities, but reflects a passive system of energy transmission and is largely anatomically determined^11,39^. The better flexibility of girls may be explained by higher body fat percentage and lower muscle mass^66,68^ and resulting lower tissue density in girls. Behavioral aspects like gender-specific sports participation might contribute to the better flexibility of girls. For instance, girls may be more frequently encouraged to participate in dance or gymnastics, while certain sports that enhance muscle tone are more popular in boys^72–74^.

In line with previous research^8,14^, third-graders’ age effects were linear in six fitness components, and this also applied to flexibility. Age gains in cardiorespiratory endurance, coordination, speed, powerLOW and powerUP were of the same size as those reported previously^8,13,14^, with the largest age gain for powerUP and the smallest for endurance. Interestingly, flexibility was the only one out of the six fitness components with a small negative age effect. This small negative age effect on stand-and-reach performance may be explained by an age-related decline in sitting/standing height ratio, i.e., an increased leg length relative to trunk length^68^. Further, age-related declines in flexibility may be associated with increases in bone length and relatively slower adjustments in muscle–tendon units^75^. In line with the assumption of growth-related changes in flexibility, performance in the sit-and-reach test was negatively associated with body height in youth aged 11 to 17 years^76^ and youth with a mean age of approximately 12 years^77^. Other studies on the development of flexibility in children and adolescents yield inconsistent results. While a cross-sectional study reported a decline of flexibility between the ages 11 and 17 years^76^, a longitudinal study assessing the fitness development in children between 9 and 12 years showed that in girls, flexibility increased linearly, whereas in boys there was no evidence for changes in flexibility during this period^12^. Other research reported no evidence for changes in flexibility between the ages 4 and 17^73^ or from second to fourth grade of primary school^58^. In contrast to the studies mentioned above, our study included data from a large sample of children within a very small age window (i.e., 7.9 to 9.6 years). Age-related changes in flexibility during this period were small and may not have been detectable in studies with smaller samples and wider age ranges.

High correlations on the child level between tests assessing cardiorespiratory endurance, coordination, speed and powerLOW, indicating the latent construct of physical fitness, as well as lower correlations between the ball-push test assessing powerUP with the other fitness tests were also replicated^8^. Correlations between performance in the stand-and-reach test and the other fitness tests were lower. As mentioned above, flexibility, unlike the other fitness components, is not energetically determined or information-oriented, but is classified as a passive system of energy transmission^11,39^. Although flexibility has been classified as a component of health-related physical fitness by Caspersen and colleagues^78^, researchers have argued that flexibility is less indicative of health than other fitness components^79,80^, and is not part of same “physical fitness” construct as tests assessing cardiorespiratory endurance, speed, muscle power/strength, and coordination^81^; it may thus be assessed with lower priority^79^. Due to its lower association with children’s health status compared to the other physical fitness tests, the stand-and-reach test was removed from the EMOTIKON test battery in 2016 and replaced by the one-legged stance test assessing static balance.

Our study has limitations. We did not use experimental data, but tested effects of assessment time, age, sex, and cohort on children’s physical fitness using quasi-experimental observational data. Due to the lack of experimental control and randomization, one must be careful when interpreting results based on observational data, especially when deriving recommendations for practice^82^. Another limitation relates to the fact that 2009 was the first cohort in which the EMOTIKON study was conducted state-wide in the Federal State of Brandenburg, Germany. Some of the performance differences between cohorts 2009 and 2010 may therefore be due to factors specifically associated with implementing the test protocol for the first time in cohort 2009, instead of due to secular physical fitness trends. If this is the case, using extrapolations of cohort effects from cohorts with assessment in second school term (2009–2010) to estimate the assessment effect may slightly over- or underestimate the effect.

When testing children’s physical fitness, timing of assessment within the school year matters. Performance in several fitness tests improved *beyond age-related development* from first to second school term. When adjusting for age, coordination, speed, and upper limbs muscle power were better in the second, compared to the first half of the school year, with boys exhibiting a larger increase of upper limbs muscle power than girls. We found no evidence of changes in cardiorespiratory endurance, lower limbs muscle power and flexibility from first to second school term. As physical fitness of age-matched children differs by time of assessment within the year, time of assessment could be considered when generating norm values and when comparing children’s physical fitness to such norms.

### Supplementary Information

Supplementary Information.

## Data Availability

Data as well as R and Julia scripts are available in the Open Science Framework (OSF) repository: https://osf.io/4vj2q/.
