# Supplementary material for: Association between time of assessment within a school year and physical fitness of primary school children
Source: Sci Rep. 2024 May 20;14:11500. doi: 10.1038/s41598-024-61038-x (PMC11106081; doi:10.1038/s41598-024-61038-x)
Supplement: Supplementary file 1 — Supplementary Information. [file 41598_2024_61038_MOESM1_ESM.pdf]

Association between time of assessment within a school year and physical fitness of primary school children

Paula Teich, Kathleen Golle, Reinhold Kliegl

Supplementary Material

Table S1 Sample description of third-graders tested in six physical fitness tests either in first (i.e., cohorts 2011–2015) or second (i.e., cohorts 2009+2010) term of school year

Gender	*Assessment*	*N Schools*	*N Children*	*Age in years Mean (SD)*	*Score Mean (SD)*
Cardiorespiratory endurance (6-min-run)					
Boys	1st school term	461	25,917	8.51 (0.29)	1050.0 m (151.3)
Boys	2nd school term	414	9,852	9.08 (0.30)	1066.8 m (151.0)
Girls	1st school term	462	26,446	8.50 (0.29)	973.3 m (130.8)
Girls	2nd school term	415	10,197	9.05 (0.31)	991.0 m (132.4)
Coordination (star-run)					
Boys	1st school term	461	25,740	8.51 (0.29)	2.08 m/s (0.30)
Boys	2nd school term	415	9,885	9.08 (0.31)	2.18 m/s (0.30)
Girls	1st school term	462	26,434	8.50 (0.29)	2.02 m/s (0.27)
Girls	2nd school term	416	10,249	9.05 (0.31)	2.11 m/s (0.27)
Speed (20-m sprint)					
Boys	1st school term	461	26,219	8.51 (0.29)	4.56 m/s (0.42)
Boys	2nd school term	416	10,080	9.08 (0.31)	4.66 m/s (0.43)
Girls	1st school term	462	26,722	8.50 (0.29)	4.43 m/s (0.39)
Girls	2nd school term	417	10,376	9.05 (0.31)	4.54 m/s (0.39)
Lower limbs muscle power (powerLOW, standing long jump)					
Boys	1st school term	461	26,395	8.51 (0.29)	129.7 cm (19.4)
Boys	2nd school term	416	10,193	9.08 (0.30)	133.9 cm (20.4)
Girls	1st school term	462	26,984	8.50 (0.29)	122.3 cm (18.5)
Girls	2nd school term	417	10,546	9.05 (0.31)	126.2 cm (19.4)
Upper limbs muscle power (powerUP, ball-push test)					
Boys	1st school term	461	26,436	8.51 (0.29)	4.0 m (0.7)
Boys	2nd school term	416	10,102	9.08 (0.30)	4.4 m (0.7)
Girls	1st school term	462	27,002	8.50 (0.29)	3.5 m (0.6)
Girls	2nd school term	417	10,455	9.05 (0.31)	3.8 m (0.7)
Flexibility (stand-and-reach test)					
Boys	1st school term	461	26,309	8.51 (0.29)	99 cm (6.8)
Boys	2nd school term	416	10,101	9.08 (0.30)	98 cm (7.0)
Girls	1st school term	462	26,961	8.50 (0.29)	102 cm (6.8)
Girls	2nd school term	417	10,538	9.06 (0.31)	102 cm (6.9)

Note. Scores of the stand-and-reach test were squared for analysis.

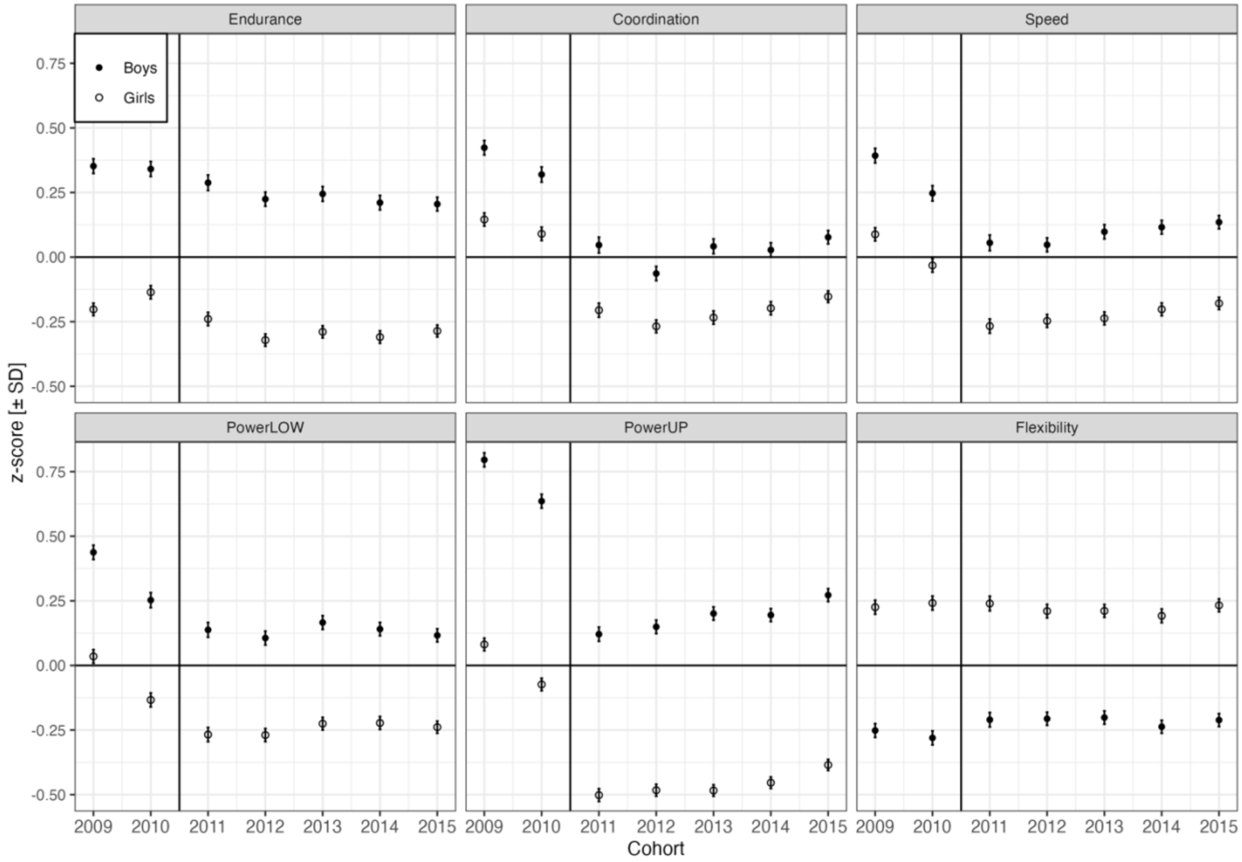

Figure S1. Zero-order physical fitness by cohort and sex. Points are mean z-scores with 95% CIs. The vertical line at 2010.5 separates cohorts with assessment in second school term (2009 and 2010) from cohorts with assessment in first school term (2011 – 2015). Endurance = cardiorespiratory endurance (i.e., 6-min run), Coordination = star-run, Speed = 20-m linear sprint, PowerLOW = lower limbs muscle power (i.e., standing long jump), PowerUP = upper limbs muscle power (i.e., ball-push test), flexibility = stand-and-reach test. Note that children tested in the second school term (i.e., cohorts 2009 and 2010) were on average a half year older than children tested in the first school term (i.e., cohorts 2011 – 2015). The superior performance in cohorts 2009 and 2010 reflects (1) the later time of assessment in the school year and (2) the older average age of these children.

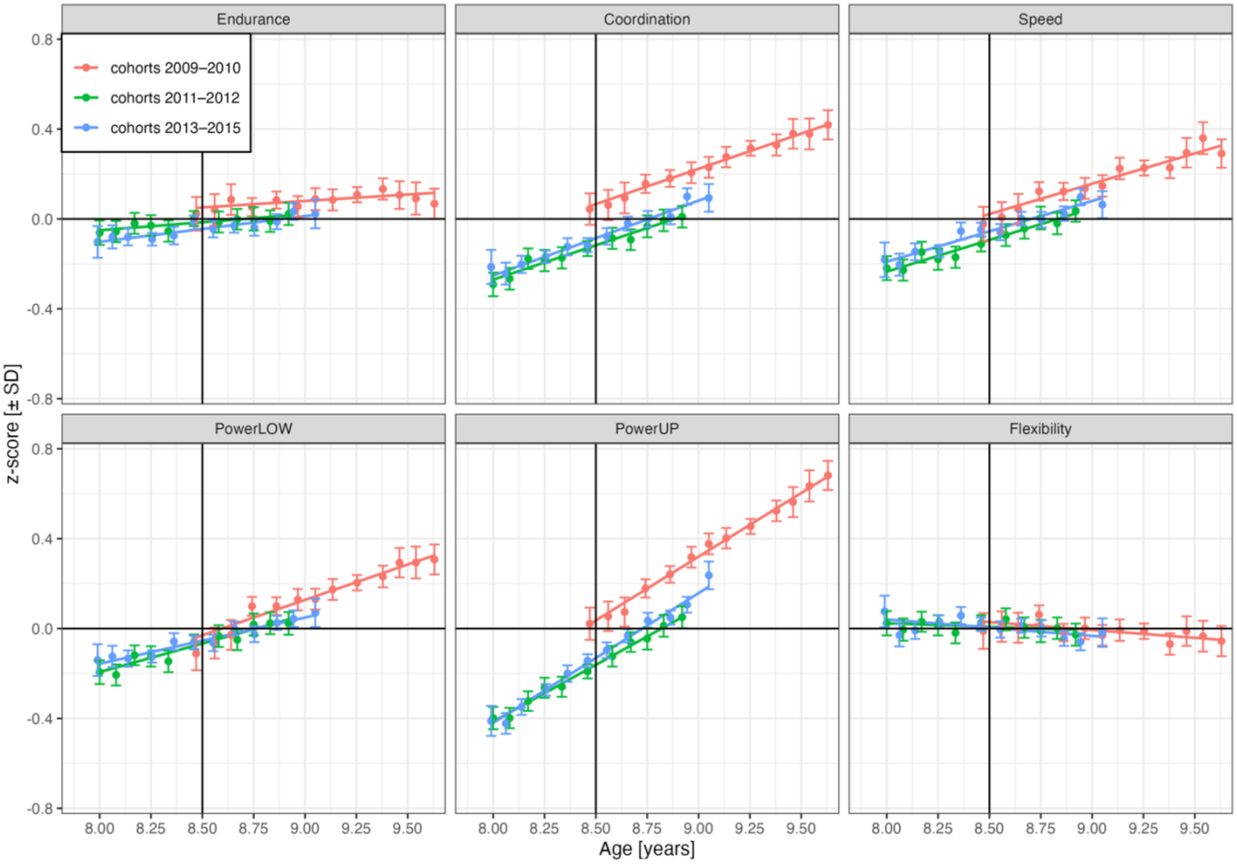

Figure S2. Physical fitness by age and cohort. Points are binned child means with 95% CIs. In cohorts 2009 and 2010, physical fitness was tested in second school term, while in cohorts 2011 until 2015, assessments were in first school term. Endurance = cardiorespiratory endurance (i.e., 6-min run), Coordination = star-run, Speed = 20-m linear sprint, PowerLOW = lower limbs muscle power (i.e., standing long jump), PowerUP = upper limbs muscle power (i.e., ball-push test), Flexibility = stand-and-reach test.

Variance components (VCs) and correlation parameters (CPs) of linear mixed model (LMM)

VCs of the random effect structure of the LMM reported in Tables 2 and 3 in the article indicated that schools differed in their assessment effects (i.e., VCs between 0.219 and 0.606), linear cohort trends (i.e., VCs between 0.009 and 0.035), and changes of linear cohort trends before and after 2010.5 (i.e., VCs between 0.139 and 0.467). There was evidence that schools with lower physical fitness at 2010.5 tended to exhibit (1) larger assessment effects and (2) larger linear cohort trends between 2011 and 2015. The following paragraphs provide a more detailed report of CPs shown in Table 3 that are related to the random factor School.

Differences between schools in their assessment effect. In line with the "law of diminishing returns", a re-parameterized LMM with physical fitness component *levels* instead of fitness component *contrasts* in the random effect structure showed smaller assessment effects for schools with higher average performance in the six fitness tests estimated in 2010.5 (CPs: cardiorespiratory endurance: -0.37, coordination: -0.48, speed: -0.46, powerLOW: -0.49, powerUP: -0.47, flexibility: -0.46; for more details, see script *Assessment.qmd* in the OSF repository). Similar correlational patterns were found between assessment effects and physical fitness contrasts (see Table 3 in article): (1) Schools with better cardiorespiratory endurance relative to CSL at 2010.5 tended to exhibit smaller assessment effects on cardiorespiratory endurance (r = -0.34), (2) schools with better coordination relative to SL tended to exhibit smaller assessment effects on coordination (r = -0.36), (3) schools with better speed relative to powerLOW tended to exhibit smaller assessment effects on speed (r = -0.37), (4) schools with better powerLOW relative to speed tended to exhibit smaller powerLOW assessment effects (r = 0.34), (5) schools with better powerUP relative to ECSL tended to exhibit smaller powerUP assessment effects (r = 0.41), and (5) schools with better flexibility relative to ECSL tended to exhibit smaller flexibility assessment effects (r = 0.42).

Differences between schools in their linear cohort trends between 2011 and 2015. Schools with lower physical fitness at 2010.5 tended to exhibit larger secular gains between 2011 and 2015. This was indicated by negative CPs between schools' performances in six physical fitness components at 2010.5 and the linear cohort trends of the corresponding physical fitness components, estimated in a re-parameterized LMM with fitness component *levels* instead of physical fitness *contrasts* in the random effect structure (CPs: cardiorespiratory endurance: -0.74, coordination: -0.81, speed: -0.76, powerLOW: -0.72, powerUP: -0.79, flexibility: -0.70). Similar correlational patterns were found in the LMM including physical fitness component contrasts in the random effect structure reported in Table 3 in the article: There were small negative CPs between schools' intercepts at 2010.5 and their linear cohort trends between cohorts 2011 and 2015 (CPs: cardiorespiratory endurance: -0.33, coordination: -0.48, speed: -0.36, powerUP: -0.30). Regarding physical fitness component

contrasts estimated at 2010.5, schools were more likely to exhibit secular (1) endurance gains between 2011 and 2015 if they exhibited lower cardiorespiratory endurance relative to CSL (r = -0.64); (2) coordination gains if they exhibited lower coordination relative to SL (r = -0.72); (3) speed gains if they exhibited lower speed relative to powerLOW (r = -0.63); (4) powerLOW gains if they exhibited lower powerLOW relative to speed (r = 0.40); (5) powerUP gains if they exhibited lower powerUP relative to ECSL (r = 0.62); and (6) flexibility gains if they exhibited lower flexibility relative to ECSL (r = 0.50).

Differences between schools in their changes of linear cohort slope before and after 2010.5 (i.e., interaction between assessment effect and linear cohort trend, Δ Cohort 2009 – 2010 [linear]). Correlations between the change of cohort trends with assessment effects or with cohort trends during 2011–2015 need to be interpreted with caution, as the correlated variables share information. Possibly, negative CPs between change in cohort slope and cohort slope between 2011–2015 are explained by the fact that schools with less pronounced secular fitness gains between 2011 and 2015 had “more room” for positive differences between cohort slopes before and after 2010.5. In fact, CPs between change in cohort slope and linear cohort trends between 2011 and 2015 were negative, with CPs ranging from -0.14 for flexibility to -0.43 for coordination. Changes in linear cohort trends before and after 2010.5 of the six physical fitness components correlated positively with assessment effects (CPs between 0.44 and 0.67), indicating that schools with larger assessment effects tended to exhibit larger (i.e., more positive) cohort trends before 2010.5 relative to after 2010.5 (see Table 3 in article).
